# Chromosome-level genome assembly of the morabine grasshopper *Vandiemenella viatica*19

**DOI:** 10.1038/s41597-024-03858-0

**Published:** 2024-09-12

**Authors:** Xuan Li, Suvratha Jayaprasad, Elisabet Einarsdottir, Steven J. B. Cooper, Alexander Suh, Takeshi Kawakami, Octavio Manuel Palacios-Gimenez

**Affiliations:** 1 Department of Organismal Biology—Systematic Biology, Science for Life Laboratory, Evolutionary Biology Centre, Uppsala University, 75236 Uppsala, Sweden; 2 Population Ecology Group, Institute of Ecology and Evolution, Friedrich Schiller University Jena, 07743 Jena, Germany; 3 Science for Life Laboratory, Department of Gene Technology, KTH-Royal Institute Technology, SE-17121 Solna, Sweden; 4 Evolutionary Biology Unit, South Australian Museum, Adelaide, SA 5000 Australia; 5 School of Biological Sciences and Environment Institute, The University of Adelaide, Adelaide, SA 5005 Australia; 6 Centre for Molecular Biodiversity Research, Leibniz Institute for the Analysis of Biodiversity Change, Adenauerallee 127, 53113 Bonn, Germany; 7 Institute of Evolutionary Biology and Ecology, University of Bonn, An der Immenburg 1, 53121 Bonn, Germany; 8 Embark Veterinary, Inc., Boston, MA USA; 9 German Centre for Integrative Biodiversity Research (iDiv) Halle-Jena-Leipzig, Puschstraße 4, 04103 Leipzig, Germany

**Keywords:** Evolutionary genetics, Evolutionary biology

## Abstract

Morabine grasshoppers in the *Vandiemenella viatica* species group, which show karyotype diversity, have been studied for their ecological distribution and speciation in relation to their genetic and chromosomal diversity. They are good models for studying sex chromosome evolution as “old” and newly emerged sex chromosomes co-exist within the group. Here we present a reference genome for the *viatica*19 chromosomal race, that possesses the ancestral karyotype within the group. Using PacBio HiFi and Hi-C sequencing, we generated a chromosome-level assembly of 4.09 Gb in span, scaffold N50 of 429 Mb, and complete BUSCO score of 98.1%, containing 10 pseudo-chromosomes. We provide Illumina datasets of males and females, used to identify the X chromosome. The assembly contains 19,034 predicted protein-coding genes, and a total of 75.21% of repetitive DNA sequences. By leveraging HiFi reads, we mapped the genome-wide distribution of methylated bases (5mC and 6 mA). This comprehensive assembly offers a robust reference for morabine grasshoppers and supports further research into speciation and sex chromosome diversification within the group and its related species.

## Background & Summary

The wingless-matchstick morabine grasshoppers within the *Vandiemenella viatica* species group (referred to as *viatica* group henceforth) constitute a distinct category of low-mobility insects that are native to southeastern Australia^1^. They primarily feed on shrubs belonging to Compositae family (*Olearia* spp., *Helichrysum* spp.). *Vandiemenella* contains two nominal species (*V. pichirichi* and *V. viatica*) and 11 provisional taxa (*viatica*17, *viatica*19, P24X0, P24XY, P24XY-translocation, P25X0, P25XY, P45bX0, P45bXY, P45cX0, and P50X0) known as chromosomal races as they can be distinguished cytogenetically and morphologically. The taxa have diverged < 3.1 million years ago from a common ancestor^2^. The observed cytogenetic differences are, however, caused by extensive chromosome rearrangements, including centric fusions, fissions, translocations, and inversions^3,4^. The *viatica* group shows a relatively limited distribution spanning from Tasmania to Eyre Peninsula in South Australia, totaling approximately 400,000 square kilometers. They demonstrate a parapatric distribution, meaning no two races coexist extensively, but rather meet in narrow regions of overlap typically ranging from 200 to 300 meters wide^4^.

The *viatica* group has been used as a model for multi-discipline research subjects. For instance, as a declining taxon, it has been used to test if population translocations can facilitate habitat restoration and be beneficial to biodiversity^5^. Scholars have used *viatica* taxa to study speciation because of their parapatric distribution patterns^1,3,6–8^. Previous studies proposed that chromosome rearrangements have contributed to hybrid dysfunction or underdominance, thereby promoting speciation^2–4^. Yet, genetic evidence for such hypotheses remains scarce due to lack of relative genomic data. The phenomenon of chromosome rearrangements within the *viatica* group provides a good model to study sex chromosome evolution, because centric fusions between the ancestral X chromosome and autosomes occurred with different autosomes in at least three instances (P24X0/XY, P25X0/XY, and P45bX0/XY), leading to evolutionarily independent cases of newly evolved neo-XY chromosomes^4^. Comparative genomics on ancestral X and neo-XY chromosomes can provide a better understanding of the evolutionary fate of “old” and “new” sex chromosomes. However, the lack of a good reference genome limits further investigation of evolution of sex chromosomes as well as the genetic basis of diversification within the *viatica* group. We thus aimed to provide a high-quality reference genome and offer relative genomic data that can facilitate studies of the *viatica* group.

Here, we report a chromosome-level annotated genome for a male of the chromosomal race *viatica*19, featuring the haploid chromosome number n = 9 + X0 (9 autosomes and one X chromosome, no presence of the Y chromosome), which is regarded as an ancestral karyotype for the *viatica* group^2^. To achieve this, we assembled a state-of-the-art backbone genome using PacBio HiFi long reads and high-throughput chromatin conformation capture (Hi-C) long-range scaffolding of a male. The assembly includes models for nine autosomes and the X chromosome. Chromosome-level assembly spans 4.09 Gb with a total of 6,634 scaffolds, scaffold N50 of 429 Mb, and BUSCO scores of 98.1%. The chromosome-level assembly was annotated with 19,304 protein-coding genes and a total of 75.21% of repetitive DNA. This chromosome-level genome assembly facilitates genomic research into the evolutionary history of speciation and sex chromosome diversification within the *viatica* group and its close relatives.

## Methods

### Sample collection

**Fig. 1:**
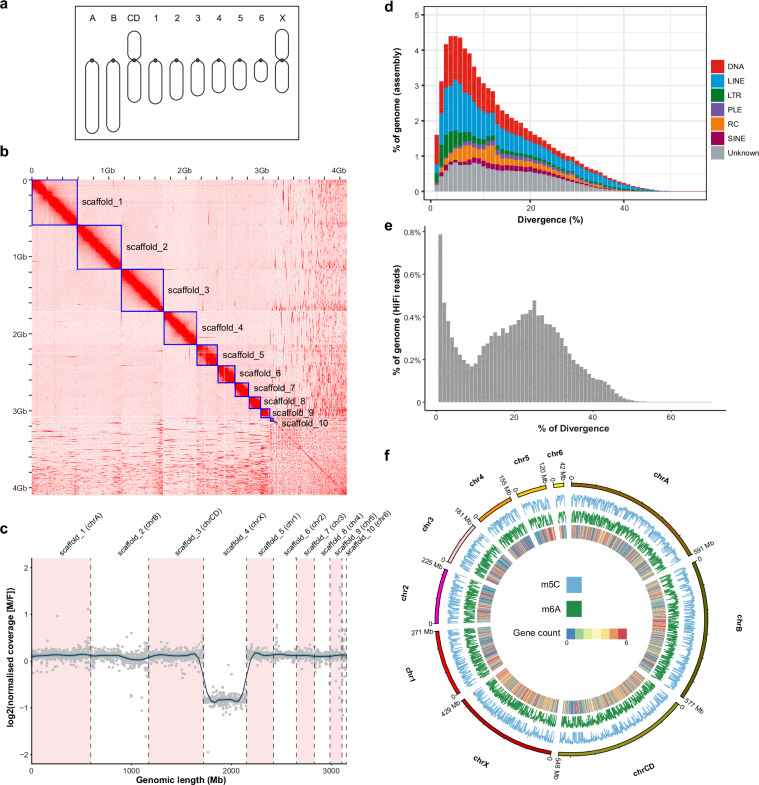
(**a**) Schematic drawing of the karyotype of chromosome race *viatica19*, according to ref. ^2^. Dots indicate positions of the centromere on each chromosome. Two pairs of acrocentric autosomes are designated as chromosome A and chromosome B. The metacentric autosome pair is named chromosome CD. Chromosome 1 to chromosome 6 are small acrocentric autosomes. The X chromosome is metacentric. Chromosomes are sorted by size and centromere position. (**b**) Hi-C contact map indicates ten super scaffolds (indicated by blue boxes), corresponding to 10 chromosomes of *viatica19*. (**c**) Distribution of male and female Illumina reads coverage ratio for 1 Mb window in each chromosomal scaffold. The log2 values close to 0 indicate that the read coverage is comparable between males and females. The values around −1 indicates the coverage in males is half of that in females. Black line represents the trend of coverage ratio distributions, and blue shading indicates 0.95 confidence interval. Reduced coverage in males compared to females in scaffold_4 indicates that this scaffold corresponds to the X chromosome. (**d**) Assembly-based TE landscape. The divergence between TE copies and their consensus sequences is shown on the X-axis as genetic distance calculated using the Kimura 2-parameter distance. The percentage of the genome assembly occupied by TEs is shown on the Y-axis. (**e**) HiFi read-based satDNA landscape. The divergence between satDNA copies and their consensus sequences is shown on the X-axis as genetic distance calculated using the Kimura 2-parameter distance. The Y-axis represents the percentage of the genome that consists of satDNA in the analyzed HiFi sequencing reads. (**f**) Genome-wide density of methylated bases (5mC and 6 mA modification) in 1 Mb windows. The heatmap in the inner circle indicates gene counts in each window. All values in the figure are logged by 2. Values for 5mC range from 0–12. Values for 6 mA range from 0–10.

Males and females of *viatica*19 were collected between 2002 and 2017 in southern Australia. The race was distinguished either through karyotyping of males or by assessing 10 morphometric characters of female genitalia, which unequivocally differentiate it from other chromosomal races^9^. Testes were dissected and fixed for karyotyping as described previously^3,10^. The remaining body parts were flash frozen in liquid nitrogen and preserved at −80 °C in the Australian Biological Tissue Collection (South Australian Museum) until DNA extraction. DNA extraction was done from either heads or legs using the Monarch HMW DNA extraction Kit (New England Biolabs, Ipswich, MA, USA; Cat No. NEB #T3010). The male haploid chromosome number for *viatica*19 was n = 9 + X0, consistent with karyotype descriptions^4^. It comprises two pairs of acrocentric autosomes designated chrA and chrB, an unequal-armed metacentric autosome pair chrCD, six pairs of small acrocentric autosomes designated chr1 to chr6, and a metacentric X chromosome (Fig. 1a).

### Genome sequencing

A total of six males and five females of the morabine grasshopper *viatica*19 were sequenced using different platforms: i) We sequenced the genome of one male using PacBio HiFi long-read sequencing (5 Sequel SMRT cells 1 M on a PacBio Sequel II system), that produced 10,159,028 HiFi raw reads (~110.81 Gb, genome coverage ~28x). Mean HiFi read length was 10,907 bp; median HiFi read quality was 14; and mean HiFi number of passes Q36; ii) one male was used to generate paired-end libraries (2 × 150 bp read length) of chromatin conformation capture (Hi-C) using the Dovetail Genomics Omni-C kit (Scotts Valley, CA, USA; Cat. No. #21005). Sequencing was done on Illumina HiSeq 2500 and produced ~445 M read-pairs; iii) we re-sequenced four male individuals and five female individuals on Illumina NovaSeq 6000 (2 × 150 bp read length) to ~20x genome coverage.

### Genome assembly and Hi-C scaffolding

We assembled the PacBio HiFi long-read sequencing into a phased primary assembled with Hifiasm v0.19.8^11^ with default parameters. Subsequently, haplotigs and contig overlaps were eliminated with Purge_Dups v1.2.6^12^. Hi-C reads were preprocessed and mapped to the primary assembly with Pairtools^13^, and the Hi-C scaffolded with YaHS v1.1^14^. A Hi-C contact map was generated with JuicerTools^15^ (v1.11.08). Finally, Juicebox^16^ (v1.11.08) was used to visualize Hi-C contact map and make manual curation for the correct boundary of the super scaffolds. The final chromosome-level assembly contains 10 chromosome models spanning 4.09 Gb in size (N50 scaffold 429 Mb), with unplaced scaffolds included (Fig. 1b; Table 1). The 10 chromosomal scaffolds comprised sequences of ~3.14 Gb in length, covering ~76.77% of the whole assembly. We evaluated the completeness of the newly generated chromosome-level assembly using BUSCO with the insecta_odb10 data (n = 1,367) in BUSCO^17,18^ (v5.5.0). This analysis revealed that the chromosome-level assembly of *viatica*19 contained C:98.1% [S:93.1%, D:5.0%], F:1.1%, M:0.8%, n:1,367 (Table 1) indicating the assembly well captured protein-coding genes. In addition, we estimated the genome size of *viatica*19 using a k-mer-based approach in GenomeScope 2.0^19^ with Illumina reads of one male individual. The k-mers in fastq.gz files were counted using squeakr v0.7^20^ with the setting “-e -k 21 -s 35 -t 16”. A list of k-mers was obtained with the command “squeakr list”, converted into a histogram using a custom script (https://github.com/octpalacios/kmer_list_to_hist), and then parsed to GenomeScope to determine the k-mer frequency distribution.

**Table 1:** Genome assembly and annotation statistics of *Vandiemenella viatica*19.

Species	*V. viatica*19
Genome size (Gb)	4.09
Scaffolds	6,634
N50 scaffold (Mb)	429
L50 scaffold count	4
Contigs	9,384
N50 contig (Mb)	1.23
Protein-coding genes	19,034
mRNA	22,150
BUSCO	C:98.1% [S:93.1%, D:5.0%], F:1.1%, M:0.8%, n:1367
TE content (%)	66.90
*SINE (%)*	3.08
*LINE (%)*	23.66
*LTR (%)*	5.54
*DNA transposons (%)*	18.38
*RC (%)*	5.51
*Unknown (%)*	16.26
Small RNA	2.58
Satellite DNA (%)	2.59 (12.26^a^)
Simple repeats (%)	0.00
Low complexity	0.10
Total repeat content (%)	75.21

a = Satellite DNA content estimated from HiFi reads with SRF^11,24^.

### X chromosome identification

In *viatica19*, the sex-determining system is a X0 system, meaning females possess two copies of the X chromosome, while males have only one copy of the X without the Y chromosome. We identified the X chromosome based on read coverage in re-sequenced reads from males and females. The analysis used Illumina re-sequenced short reads of five individuals per sex and the newly generated chromosome-level assembly. Individuals of *viatica*19 were mapped separately onto the chromosome-level assembly, using BWA-MEM^21^ (v0.7.17) with default parameters. The output bam files were sorted into coordinate order, and duplicates were removed using SAMtools^22^ (v1.14) sort and markdup function. The coverage of 1 Mb windows was calculated for each chromosomal scaffold with mosdepth^23^ (v0.3.3) and then normalized by the average genomic coverage of each individual. We calculated the ratios of male and female coverage for each window and plotted the log2(ratio) using ggplot2^24^ (v3.4.4). While other scaffolds show the log2(ratio) around 0 indicating similar coverages in males and females, the log2(ratio) numbers of scaffold_4 are mainly around −1 (Fig. 1c), indicating the male coverage of the scaffold is half of the female coverage. Thus, scaffold_4 represents the assembly of the X chromosome. The remaining 9 chromosomal scaffolds were preliminarily designated as chrA, chrB, chrCD, chr1-6 respectively, according to the size differences indicated by the karyotype from previous studies^2^. Note that further validation is necessary for more accurate allocations of *viatica*19 autosomes.

### Repeat annotation

We used Satellite Repeat Finder^25^ (SRF, v1.0) to identify motifs in satellite DNA (satDNA) that were tandemly arranged in the PacBio HiFi reads. Additionally, we employed RepeatModeler2^26^ (v2.0.4) to *de novo* predict repetitive elements in the new chromosome-level assembly and construct a repeat library. The SRF and RepeatModeler2 libraries were merged using ReannTE (source code: https://github.com/4ureliek/ReannTE/tree/master) with the flag “-s 80”, and given preference setting to repeat libraries derived from chromosome-level assemblies when selecting consensus sequences to retain. Consensus sequences resembling proteins in the library were filtered using the workflow named Repeat library filtering (source code: https://github.com/NBISweden/repeatlib_filtering_workflow). The filtered library was subsequently merged with Arthropoda consensus sequences from Repbase^27^. This final library was then used to annotate the chromosome-level assembly with RepeatMasker v4.1.0^28^. The annotation was processed with the script calcDivergenceFromAlign.pl from RepeatMasker utils to calculate the divergence between repeats and their consensus sequences using the Kimura 2-parameter distance corrected for the presence of CpG sites (Fig. 1d). The assembly was annotated with 75.21% of repetitive elements, mainly including 23.66% LINE, 18.38% DNA transposons, 5.54% LTR, 5.51% rolling-circles (RC), 2.59% satDNA and 16.26% unclassified repeat sequence (Table 1). The repetitive DNA content of *viatica*19 (75.21%) is comparable to those of other related morabine grasshoppers (i.e. P24X0/XY, P45bX0/XY) previously studied, ranging from 71% to 80%^29^. The genome of *viatica*19 shows instances of amplification of TE copies throughout the genome. Notably, a recent TE burst has been observed within the 0–10% divergence range (Fig. 1d).

We further quantified satDNA abundance in the PacBio HiFi reads. To estimate relative genomic abundance and nucleotide divergence (Kimura 2-parameter distance) for each satDNA, 500,000 PacBio HiFi reads were sampled and aligned to the SRF satDNA library using RepeatMasker. The resulted alignment file was then parsed to the script calcDivergenceFromAlign.pl from RepeatMasker utils. The relative abundance of satDNA was represented as the proportion of aligned nucleotides with respect to the overall size of PacBio HiFi reads, which was 12.26% (Fig. 1e). This number is much higher than the 2.59%, which is the proportion of satDNA detected in the assembly, indicating that satDNA sequences are among the most collapsed repeats in the assembly (Table 1). The satDNA repeat landscape reveals two episodes of repeat amplification. The first burst is relatively recent, occurring in the divergence range of 0–5%, while the second burst is older, occurring in the 15–25% divergence range (Fig. 1e).

### Genome annotation

The repeat-masked genome was used for the gene model annotation with the GeMoMa^30^ v1.9 pipeline. GeMoMa leverages the annotation of protein-coding genes in a reference genome to infer the annotation of protein-coding genes in a target genome, using both amino acid sequence and intron position conservation. Moreover, GeMoMa offers the option to integrate RNA-seq evidence for splice site prediction. We used previously published RNA-seq reads from males and females of closely related chromosomal race P24X0^29^ (BioProject PRJNA668746) to assist in gene prediction. Paired-end RNA-seq reads of P24X0 individuals were first aligned to the repeat-masked genome with HiSat2 v2.2.1^31^ with the “–dta” parameter on default setting. The resulting BAM alignment file was then sorted with SAMtools v1.14 and used to run GeMoMa in conjunction with *Drosophila melanogaster* Release 6^32^ (GCA_000001215.4), *Caenorhabditis elegans*^33^ (GCA_000002985.3), *Gryllus bimaculatus*^34^ (GCA_017312745.1), *Schistocerca gregaria*^35^ (GCA_023897955.2), *Tribolium castaneum*^36^ (GCA_000002335.3) and *Daphnia pulex*^37^ (GCA_900092285.2) genome and annotation files as references for the homology-based gene prediction in the assembly. A total of 19,034 protein-coding genes was predicted in the chromosome-level assembly (Table 1). Functional annotation of protein-coding genes at a genome-wide scale was evaluated based on InterProScan^38^ v5.52–86.0. The number of predicted protein-coding genes in *viatica19* (19,034) is comparable to that observed in the desert locust grasshoppers *Locusta gregaria* (17,307)^39^ and *Schistocerca gregaria* (18,815)^40^.

### Genome-wide base modification distribution

We detected base modification in the genome of *viatica*19 using the PacBio HiFi long reads. The reads were initially mapped to the newly generated chromosome-level assembly with pbmm2 (https://github.com/PacificBiosciences/pbmm2.git) from the SMRT tools (v13.0) with default parameters. The aligned reads were then used to call for the base modification signatures such as 5mC and 6 mA using ipdSummary function in SMRT tools. For each assembled chromosome, the total percentages of methylated bases ranged from ~4.7% (chrCD) to ~10.9% (chr6) (Table 2). Wherein, average percentages of 5mC per 10 Mb ranged from ~2.7% (chrCD) to ~5.1 (chr6) among the ten chromosomes. For 6 mA, average percentages per 10 Mb ranged from ~0.7% (chrCD) to ~1.1% (chr4), indicating differences in methylated base types. To demonstrate genome wide distribution of methylated bases, we plotted numbers of 5mC and 6 mA bases in 1 Mb windows in comparison to gene counts (Fig. 1f).

**Table 2:** Total numbers and percentages of methylated bases on each chromosomal scaffold.

Scaffold	No.of bases (Mb)	No. of m5C (Kb)	No. of m6A (Kb)	Total no. of m5C + m6A (Kb)	% of m5C (Kb)	% of m6A (Kb)	% of m5C + m6A (Kb)	No. of genes methylated
scaffold_1	591.11	207.72	106.39	314.11	3.51%	1.80%	5.31%	2968
scaffold_2	577.52	196.94	96.69	293.63	3.41%	1.67%	5.08%	2708
scaffold_3	548.11	173.69	85.07	258.76	3.17%	1.55%	4.72%	2604
scaffold_4	429.17	140.26	73.40	213.66	3.27%	1.71%	4.98%	1603
scaffold_5	271.38	111.80	55.92	167.71	4.12%	2.06%	6.18%	1418
scaffold_6	225.05	94.59	46.89	141.48	4.20%	2.08%	6.29%	1332
scaffold_7	180.57	90.59	43.31	133.89	5.02%	2.40%	7.41%	1159
scaffold_8	155.37	84.58	40.52	125.10	5.44%	2.61%	8.05%	1146
scaffold_9	120.41	63.45	27.23	90.68	5.27%	2.26%	7.53%	701
scaffold_10	41.72	24.30	20.99	45.29	5.82%	5.03%	10.85%	225

## Data Records

The raw sequencing data, the generated assembly and the annotation file are available on the NCBI database under the project with accession number PRJNA1111711. The assembly is deposited on NCBI GenBank under accession number JBFBPN000000000^41^. Raw sequencing data are deposited on NCBI Sequence Read Archive (SRA). PacBio HiFi raw reads with kinetics information are available under accession number SRX25396123^42^. The Hi-C sequencing data are available under accession number SRX24553415^43^. Illumina reads of four male and five female individuals generated from this study are deposited under accession numbers SRX24553406-SRX24553414^44–52^. Illumina reads of a male individual are obtained from previously generated data available on NCBI SRA (accession number: SRX19754992^53^). The gene annotation file and the RepeatMasker annotation file are available on the figshare database^54,55^.

## Technical Validation

The assembly was evaluated using BUSCO with the insecta_odb10 data which resulted in a complete BUSCO score of 98.1%. We estimated genome size to be ~3.86 Gb using k-mer based approach with Illumina reads, which is close to the assembly size of ~4.09 Gb. The total length of 10 pseudo-chromosomes is ~3.14 Gb, which represents ~81.35% of the estimated genome size. In addition, we mapped the Illumina re-sequenced reads to the chromosome-level assembly using BWA-MEM^21^ (v0.7.17), achieving an alignment rate of 100%. We further mapped the PacBio HiFi reads to the 10 assembled pseudo-chromosomes with Minimap2^56^ (v.2.24), achieving an alignment of 99.56%. These numbers suggest that although some of the sequences are not allocated to the pseudo-chromosomes, our chromosomal level assembly indeed captures the most information of the *viatica*19.

## Data Availability

This work did not develop novel scripts. All commands and pipelines for data processing were executed following the manual and protocols of the respective bioinformatics software. Default parameters were applied unless otherwise stated in the Methods section above.
